# High-dimensional omics data analysis using a variable screening protocol with prior knowledge integration (SKI)

**DOI:** 10.1186/s12918-016-0358-0

**Published:** 2016-12-23

**Authors:** Cong Liu, Jianping Jiang, Jianlei Gu, Zhangsheng Yu, Tao Wang, Hui Lu

**Affiliations:** 1 Department of Bioengineering, University of Illinois at Chicago, Chicago, IL USA; 2 SJTU-Yale Joint Center for Biostatistics, Shanghai Jiao Tong University, Shanghai, China; 3 Department of Bioinformatics and Biostatistics, College of Life Science, Shanghai Jiao Tong University, Shanghai, China; 4 Center for Biomedical Informatics, Shanghai Children’s Hospital, Shanghai, China

**Keywords:** Variable selection, Dimension reduction, Sure independence screening, Knowledge integration, SKI

## Abstract

**Background:**

High-throughput technology could generate thousands to millions biomarker measurements in one experiment. However, results from high throughput analysis are often barely reproducible due to small sample size. Different statistical methods have been proposed to tackle this “small n and large p” scenario, for example different datasets could be pooled or integrated together to provide an effective way to improve reproducibility. However, the raw data is either unavailable or hard to integrate due to different experimental conditions, thus there is an emerging need to develop a method for “knowledge integration” in high-throughput data analysis.

**Results:**

In this study, we proposed an integrative prescreening approach, SKI, for high-throughput data analysis. A new rank is generated based on two initial ranks: (1) knowledge based rank; and (2) marginal correlation based rank. Our simulation shows the SKI outperforms other methods without knowledge-integration in terms of higher true positive rate given the same number of variables selected. We also applied our method in a drug response study and found its performance to be better than regular screening methods.

**Conclusion:**

The proposed method provides an effective way to integrate knowledge for high-throughput analysis. It could easily implemented with our provided R package named SKI.

## Background

The understanding of the molecular basis of complex diseases such as cancer has been greatly enhanced in present time by genomic sequencing and other omics-approaches. Genomic biomarkers have been applied to disease screens [1–3], cancer subtype classification [4–6], and to predict drug response [7–9]. As large numbers of biomarkers can be measured simultaneously at a relative small cost, the bottleneck for such omics studies has become the expansion of the number of samples collected. Unfortunately, for many current studies, the number of subjects is much smaller than the number of genetic markers measured, which has ranged from thousands of genes to millions of genetic variants. Thus how to identify the relevant variables or biomarkers precisely in a high-dimensional data set has become a challenge for the further advancement of the development of precision medicine and personalized treatment.

Traditionally, variables were identified by univariate analysis, followed by multiple-testing adjustment such as Bonferroni’s *p* value correction or false discovery rate (FDR) procedure [10, 11]. For example, in genome-wide association studies (GWAS), single nucleotide polymorphisms (SNPs) are screened site-by-site to test the association between diseases and complex traits. However, this approach ignores the underlying correlation structure between genomic markers, leading to the absence of identification of the joint impacts of biomarkers on phenotypes. To address the joint impacts, popular variable selection methods such as LASSO [12], adaptive LASSO [13], and SCAD [14] have been established over the past decades. Such methods, however, are beset with high computational costs when p is as large as an exponential of the sample size n. To overcome these high computational costs in analyzing such ultra-high dimensional data, an effective solution is to conduct pre-screening of variables. For example, Fan and Lv proposed the sure independence screening (SIS) approach in which prescreening is based on marginal correlations [15]. Tibshirani et al. proposed a method to prescreening-based on a LASSO penalization under the Cox model [16].

Another way to tack this “large p small n” paradigm is to collect multiple datasets (i.e., increase n). One popular approach is to pool datasets together and then perform further analysis as if they originated from a single study. This approach demands the data to be fully comparable and it’s often not feasible to integrate datasets from different sources of genomic information. Other data integration methods have been developed by incorporating hierarchical and network-based models to integrate different omics data. Shen et al. proposed an iCluster approach to assign cancer subtype by integrating multiple levels of omics data with introducing a latent variable [17]. Aure et al. identified in‑trans process associated genes in breast cancer by integrated analysis of copy number and expression data [18]. Akavia et al. identified driving cancer mutations and the processes that they influence by integration of copy-number variation and gene expression [19]. In a recent NCI-DREAM challenge, various integration methods, such as Bayesian multitask multiple kernel learning (MKL), have been applied to identify biomarkers for drug response [20].

Such methods however are often associated with a few problems. First, most of them are very complex and sometimes difficult to apply without possession of specialized statistics knowledge. Secondly, since these methods may be designed for specific cases, they are potentially inflexible and hard to modify in order to apply to another study. Lastly, and most importantly, all of them require the access to the raw data, which often is unavailable.

The goal of this study is to develop a general procedure for variable selection with knowledge integration. The basic idea of our method is to guide the pre-screening procedure by taking prior knowledge into account, and then after prescreening, sophisticated variable selection techniques such as LASSO could be applied.

The only input required for our method is a rank of genomic biomarkers obtained from external information, which is certainly a desirable feature for the users without accessibility to raw data. For example, in one possible application, summary statistics of psychiatric disorders could be found at the Psychiatric Genomics Consortium (PGC) website [21, 22] and used to develop a ranking. This ranking could be then applied to pre-rank the SNPs in GWAS studies related to psychiatric disorders. In other applications, an association between genes and other biological terms could be obtained through text mining of the literature [23, 24], and genes could be ranked based on this association. Similarly, the genes reported to have interaction with a drug or compound [25] can be placed on the top of the list (prioritized) when predicting drug response. in the top of lists when predicting the drug response. More commonly, a candidate list could already exist before the high-through measurement procedure takes place and it is then reasonable to give these candidates a higher priority. In the most extremist case, only candidate biomarkers were measured (e.g., customized array, target sequencing or exome sequencing) instead applying a genome-wide measurement. To distinguish our method from others, we call this “knowledge integration”.

A simulation study was conducted to examine the performance of our method. We also compared it to the other popular approaches. We then applied our method in a drug response analysis. Our method outperformed a commonly used marginal correlation based screening procedure.

## Method

### Sure independence screening

Suppose we have a genomic dataset (*y*_*i*_, ***x***_***i***_), where *y*_*i*_ is the response and ***x***_***i***_ = (*x*_*i*1_, *x*_*i*2_, …, *x*_*ip*_) is the vector of p covariates, for *i* = 1, 2, …, *n*. In real applications, Y could be measurements of some phenotypes or quantitative traits, such as weights, drug response, etc. X could be some high-dimensional omics-measurements, such as gene expression, CpG methylations, etc. In a typical genomic setting, p could be far larger than n. To deal with high dimensionality, effective variable selection techniques are required.

The sure independence screening (SIS) method introduced by Fan and Lv [15] is a two stage approach. First, it selects significant predictors by sorting the corresponding marginal likelihood (correlation in linear model), thus fast reducing the ultra-high dimensionality to a relatively large scale *d* (e.g., *o*(*n*)). Subsequent to SIS, a more sophisticated lower dimensional model selection technique such as SCAD [14], the Dantzig selector [26], LASSO [12], or adaptive LASSO [13] could be applied to perform the final variable selection and parameter estimation. Apparently, SIS could dramatically speed up variable selection when the p is extremely large. Fan and Lv proved SIS enjoys the sure screening property and model selection consistency under certain conditions.

### Screening with prior knowledge integration

**Fig. 1:**
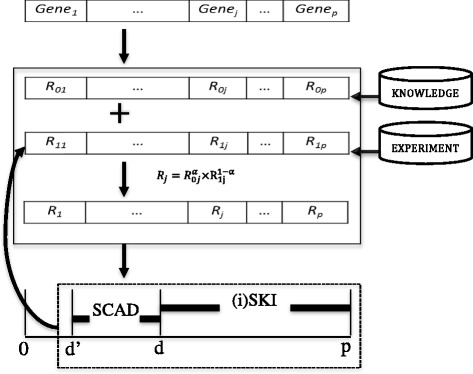
A brief description of (i)SKI procedure. For each variable, two ranks are generated, one based on prior knowledge (*R*_0_), the other based on marginal correlation (*R*_1_). A predefined α, (or estimated based on the dev. ratio) is used to control the weight of prior knowledge. Variables are then sorted by weighted geometric mean of two ranks. SKI first reduces the variable number from p to d, and then a more sophisticated method such as SCAD is used to further refine the model to size d ’ and estimate the parameters. iSKI updates the marginal correlation based rank (*R*_1_) by regressing residues over the rest p − d ’ variables. The procedure is repeated until the desired number of parameters obtained

We noted that the idea of SIS is based on marginal correlation to first select important variables. Based on this idea, we proposed an novel approach, screening with prior knowledge integration (SKI), to select variables in the first stage. The basic procedure of SKI is drawn in Fig. 1. The idea of the SKI is to rank the variables not only based on marginal correlation but to also incorporate external information. The rationale here is that the variables supported by both marginal correlation and external information are more likely to be important features, and thus should be included in the second stage for parameter estimation with larger probability.

Besides the same settings in SIS, now suppose we also have an external ranking of variables *R*_0_, which is of length *p*, obtained from prior knowledge. We define a new rank for gene *j* as the weighted geometric mean of two ranks:

$$ {R}_j={R}_{0j}^{\alpha}\times {R}_{1j}^{1-\alpha } $$

for *i* = 1, 2, …, *p. R*_0*j*_ is the rank of gene *j* obtained from prior knowledge, and *R*_0*j*_ is the rank of gene *j* obtained by sorting marginal correlation. Here *α* is a parameter controlling the importance of prior knowledge. Here, we restrict 0 < *α* < 0.5 to limit the influence of prior knowledge so that it could not be stronger than the data at hand and we will estimate it by data (introduced next). But in practice, *α* could be a value, in range from 0 to 1, predetermined by users or estimated by data. If we set *α* = 1, the genome-wide measure becomes the targeted-region measure.

The initial ranking represents the importance of each variable known ahead of the ongoing study. For example, if the goal of this study is to predict drug response based on gene expressions, other genetic measurements such as copy number variants (CNV) might be available. We could first rank each CNV by its marginal correlation with drug response obtained by univariate linear regression and then we map CNV ranks back onto the genes to get an initial rank of genes. More commonly, we could rank genes based on their importance scores obtained by expert domain knowledge or literature searching.

Typically, we require that each variable has an initial rank. For those variables with no information, an average rank can be assigned. For instance, among 100 predictors, 10 of them are found associated with response from existing knowledge. We could assign ranks (ranged from 1 to 10) to these 10 predictors based on their association strength and 55 for the rest. Alternatively, if we don’t know the association strength, we could set the ranks of 10 predictors as the average of 1 to 10, which is 5.

### Estimation of ***α***

As mentioned above, the selection of *α* could control the relative strength of influence imposed by prior knowledge, which is essential for the success of the proposed methods. Unfortunately, there is no pleasant way for tuning this parameter. LASSO or elastic-net [27], uses cross-validation strategy to select *α* with lowest internal prediction errors. However, the problem we face here is a ultra-high dimensional problem, where the number of covariates *p* is already much larger than sample size *n*. Cross validation will require us to further spit the sample into training and testing, which can make the ultra-high dimensionality issue worse. To alleviate these concerns, we develop the following alternative strategy.

We first generate a sequence of *α* = (0, 0.1, 0.2, 0.3, 0.4, 0.5). For each *α*, we re-rank the variables as its weighted geometric mean rank. We then select the top *d* ranked variables as inputs for a ridge regression model [28]. After fitting a penalized ridge regression, we calculate the fraction of null deviance explained as.

$$ dev.ratio=1-\frac{loglik{e}_{sat}- loglik e}{loglik{e}_{sat}- loglik{e}_{null}} $$

Here *loglike*_*sat*_ refers the log-likelihood or the saturated model (i.e., a model with a free parameter per observation). And *loglike*_*null*_ refers to the intercept model. We compare the *dev. ratio* across different *α*’s, and select the *α* yields largest *dev. ratio* as the final *α*.

The rationale of this method is that if one set of variables is more biologically meaningful than the other, the better it could fit a ridge regression model. We do notice that the number of variables selected *d* will affect the performance of SKI in terms of estimation of α. In the most extreme case, if only one variable is selected (*d* = 1), then the estimated α will always be zero. But our experiences suggest the number of variables selected won’t affect the results significantly if this number is not too small. Although some methods have been proposed to tune this parameter [29], how to determine the number of variables is out of the scope for this study.

### Extension: iterative SKI

Fan and Lv demonstrated that when too many predictors are involved, the basic sure screening methods might miss some important variables due to collinearity issues. In their paper they developed an iterative version of SIS to use fully the joint information of the covariates rather than marginal information. Briefly, in the first step, a subset of *k*_1_ variables is selected using an SIS-based method. Next, a *n* -vector of residuals are obtained from regressing the response *Y* over *k*_1_ variables are treated as new responses and the same method is applied to the remaining *p* − *k*_1_ variables. The process is repeated until desired number (e.g., *d*) of variables is selected or (predefined) maximum iteration is reached.

We extend this idea to SKI and developed an iterative version of SKI (iSKI). The similar procedure was used. In the first step, the rank of each variable is obtained as weighted geometric mean of knowledge-based rank and the sorting marginal correlation between responses and predictors. For the rest of the steps, the rank is weighted geometric mean of the knowledge-based rank and the sorting marginal correlation between residuals and predictors.

## Results

### Simulations

We adopted a similar simulation in Ma 2012 [30]. In total *n*_*x*_ = 200 samples (X, *Y*_*x*_) were simulated, with gene number *p* = 10, 000. 200 clusters were simulated independently, and 50 genes in each cluster were simulated from a multivariate normal distribution with *μ* = 0, *σ* ^2^ = 1 and AR(1) correlation structure *ρ* = 0. 6. (i.e., *cor*(*i*, *j*) = *ρ* ^|*i* − *j*|^). This is to mimic a real gene expression studies with taking pathway structure into account. In each cluster, the coefficients *β*’s of first ten genes were simulated from a uniform distribution with minimum 0.5 and maximum 1. All other *β*’s were set to be zeros. This is consistent with the parsimonious assumption that only few genes and pathways were associated with phenotypes or diseases. Continuous responses were generated from linear regression models with *σ*_*x*_^2^ = 1 (or 3).

Another *n*_*z*_ = 200 samples (*Z*, *Y*_*z*_) with gene number *p* = 10, 000 were simulated to mimic an external gene expression study, where our prior knowledge was drawn from. Gene expressions and responses were simulated from the same structure as described above. But the non-zero coefficients *β* were simulated to have 0, 50, and 100% overlap with non-zero *β* in the internal settings. This is to mimic the situation that the prior knowledge completely disagrees, partially agrees and exactly agrees with our true experiment settings.

To better evaluate the performance of the proposed approach, we also consider other alternatives:

1. Select genes without external knowledge available. Genes were based on marginal correlations between X and *Y*_*x*_. (SIS)
2. Select genes based on the proposed methods, where the prior ranks of genes generated based on marginal correlation between Z and *Y*_*z*_. (SKI)
3. Select genes based on pooling two dataset together and conduct analysis as one dataset. Genes were ranked based on marginal correlations. (P)

In Table 1, we summarize the results of variable selection by generating 100 datasets. As expected, under the same settings of *ρ*, *σ*_*x*_^2^, and *σ*_*z*_^2^, the estimated *α* was increased as the percentage of non-zero *β* that overlapped between internal and external datasets increased. The proposed methods selected consistently more true positive genes when prior knowledge partially or exactly agrees with internal settings (i.e., 50, 100%). When the prior knowledge is completely noisy (i.e., 0%), the performance of the proposed methods is comparable with only using an internal dataset. Although, the performance of pooling two datasets is better than the proposed methods when the prior knowledge is useful, the performance will drop dramatically when the prior knowledge is not useful. More importantly, as stated before, the focus of this study is to develop a strategy to integrate biological knowledge. Obviously, the applied range of the proposed methods is much broader.

**Table 1:** Simulation results compared the number of true positives among different methods

Positive^a^				1%			5%			10%		
%^b^	*σ*_*x*_^2^ ^c^	*σ*_*z*_^2^ ^d^	*α* ^e^	SIS^f^	SKI^g^	P^h^	SIS	SKI	P	SIS	SKI	P
0.0	1	1	0.075	38.96	38.94	36.36	45.78	45.72	43.63	47.66	47.63	45.63
0.5	1	1	0.275	38.53	43.06	45.22	45.66	47.65	48.54	47.53	48.85	49.13
1.0	1	1	0.384	38.5	46.34	47.99	45.65	48.9	49.58	47.49	49.51	49.83
0.0	1	3	0.090	39.10	38.97	35.01	45.81	45.80	42.94	47.71	47.72	44.03
0.5	1	3	0.249	38.92	42.55	43.85	45.80	47.31	48.28	47.57	48.55	49.10
1.0	1	3	0.368	39.04	45.81	47.58	45.88	48.60	49.44	47.65	49.21	49.73
0.0	3	1	0.113	36.84	36.43	35.77	44.61	44.01	43.37	46.69	46.57	46.19
0.5	3	1	0.261	37.27	42.16	44.90	45.15	47.36	48.34	47.07	48.56	49.03
1.0	3	1	0.374	36.91	46.01	48.89	44.76	49.42	49.51	47.12	49.86	49.90
0.0	3	3	0.104	37.84	37.48	35.19	45.73	45.43	44.07	47.63	47.53	45.93
0.5	3	3	0.264	37.26	42.52	44.48	45.03	47.35	48.26	47.19	48.58	49.00
1.0	3	3	0.355	37.05	45.20	47.37	45.1	48.6	49.39	47.05	49.36	49.76

^a^Top 1, 5 and 10% variables were selected respectively under different settings

^b^the percentage of non-zero *β*’s overlapped with each other in two datasets

^c^ *σ*_*x*_^2^ : the variance added in internal dataset to generate response *Y*_*x*_

^d^ *σ*_*z*_^2^: the variance added in external dataset to generate response *Y*_*z*_

^e^ *α*: the estimated value of *α* which control the weight of two ranks in geometric mean

^f^SIS: variables were sorted by marginal correlation using only internal dataset

^g^SKI: variables were sorted by weighted geometric mean of two marginal correlation based ranks using two dataset

^h^Pool: two dataset were pooled together and treated as a single dataset, and then variables were sorted by marginal correlation

We also investigated the performance of the extension of the proposed approach (iSKI), by compared it with non-iterative version of the proposed approach (SKI), SIS and iSIS methods. The last two methods were proposed by Fan 2008 to select important variables without considering prior knowledge. The extension methods were proposed to solve the issue of strong collinearity between genes. So we simulated different *ρ* (0.3 and 0.6) to investigate its performances under different correlation settings. Since both iSIS and iSKI are very computation intensive, we fixed *σ*_*x*_^2^ = 1 and *σ*_*x*_^2^ = 1. We also set the maximum iteration to three to reduce computing time. SCAD was used to fit the model in the second stage. All the other settings were kept the same as before. Table 2 summarizes the number of true positives when the top 1% genes were selected. As expected, iSIS included more true variables than SIS, and iSKI performs even better than iSIS when the external information are useful.

**Table 2:** Simulation results compared the number of true positives among iterative and non-iterative approaches when top 1% variables were selected

%^a^	*ρ* ^b^	*α* ^c^	SIS^d^	SKI^e^	iSIS^f^	iSKI^g^
0	0.3	0.061	23.32	23.12	25.22	22.53
0.5	0.3	0.342	24.83	33.20	26.13	34.43
1	0.3	0.443	23.14	34.41	26.33	38.85
0	0.6	0.044	37.35	36.34	41.11	36.17
0.5	0.6	0.392	36.47	41.67	39.67	44.83
1	0.6	0.453	37.12	45.83	40.44	49.40

^a^%: the percentage of non-zero *β*’s overlapped with each other in two datasets

^b^ *ρ*: correlation coefficients between two neighbor variables in each cluster

^c^ *α*: the estimated value of *α* which control the weight of two ranks in geometric mean

^d^SIS: variables were sorted by marginal correlation using only internal dataset

^e^iSIS: iterative version of SIS

^f^SKI: variables were sorted by weighted geometric mean of two marginal correlation based ranks using two dataset

^g^iSKI: iterative version of SKI

### Real application: drug response analysis

We next applied the SKI procedure to a drug response study and compared it to the results obtained with the SIS procedure. Selumetinib (AZD6224) is a drug used to treat various types of cancer such as non-small cell lung cancer (NSCLC). It is a potent, highly selective MEK1 inhibitor. Unfortunately, despite intensive studies, the genetic mechanism for Selumetinib resistant remains controversial [31–34]. We applied the SKI procedure to identify the potential biomarkers of response to Selumetinib. We downloaded the drug response data (i.e., Active Area) from the Cancer Cell Line Encyclopedia (CCLE) project [35] together with its baseline omics measurement, which includes gene expression, mutation data, and copy numbers. In total there were 489 cell lines and 41,872 genomic features measured. For a single feature, we assign a specific gene annotation on it. We then searched the Drug2Gene database [25] to acquire prior knowledge of association between selumetinib and genes. Drug2Gene is an integrative knowledge base reporting relations between genes/proteins and drugs/compounds including bioactivity data where available. The data has been collected from 23 public databases and integrated to provide a 'one-stop shop’ for identifying tool compounds for genes or finding all known targets of a drug. In total, 383 genes were identified to have associations with selumetinib. We gave an initial rank to 41,872 genomic features based on whether its annotated genes have a known association with selumetinib. For 1105 features with annotated genes having association with selumetinib, we set their ranks as 553, and for others, we set the ranks as 21,489.

The SKI and SIS procedure were used for variable selection, respectively. The top 100 features were selected and SCAD was used to fit the final model. In other studies, external information (e.g., biological relevance) are used to judge whether the variables identified are accurate. Since here we already used this knowledge in SKI, it is unfair to judge the results by this criteria. So we used leave-out-out cross validation (LOOCV) to compare the prediction squared error of these two methods.

**Fig. 2:**
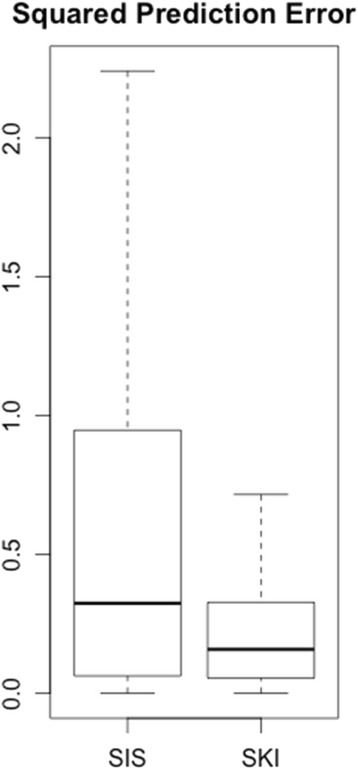
Boxplot of squared error for selumtinib response prediction using two methods. Whiskers indicate min/max, upper box limit 75% percentile, low box limit 25% percentile and line the median

The average of *α* estimated in SKI was 0.382, indicating that the prior known associated genes are very informative in variable selection. In Fig. 2, we showed the LOOCV prediction square error of two methods. In general SKI methods outperforms SIS in terms of small prediction error. The median (mean) prediction square errors are 0.324 (0.828) and 0.158 (0.397) for SIS and SKI, respectively. By integrating prior known information, SKI selects the variables more accurately.

We also investigated the features identified by these two methods. Those features identified by SKI procedure, with known association with selumtinib ahead, are summarized in Table 3. The mean absolute value of marginal correlation for all variables is 0.056, while this number increases to 0.225 for variables with previous known association. Despite the fact that genes with known association with selumtinib were highly enriched in the top of the ranked list generated by marginal correlations, only one variable, mutation of BRAF, could be recruited by using common marginal correlation based screening methods when top 100 variables were selected. But by applying the SKI procedure, we rescued 17 variables whose marginal correlations are not high enough, but supported by external knowledge in our final model.

**Table 3:** 18 variables selected by SKI procedure when top 100 variables were selected, whose association with selumetinib could be found in database

Gene Symbol	Probe ID	Type	*R*_*SIS*_^a^	*R*_*SKI*_^b^
BRAF	NA	Mut	4	1
ADCK3	56997_at	Exp	172	5
TESK1	7016_at	Exp	194	6
DCLK2	166614_at	Exp	196	8
TNIK	23043_at	Exp	206	9
NUAK2	81788_at	Exp	209	10
ERBB3	2065_at	Exp	328	14
PRKCD	5580_at	Exp	338	15
MYLK	4638_at	Exp	479	20
MAP3K1	4214_at	Exp	502	21
ULK3	25989_at	Exp	519	23
FGFR1	2260_at	Exp	556	25
SNRK	54861_at	Exp	582	26
RPS6KA3	6197_at	Exp	623	29
STK10	6793_at	Exp	691	31
MAPK9	5601_at	Exp	756	34
TAOK3	51347_at	Exp	761	35
PIK3CB	5291_at	Exp	764	36

^a^ *R*_*SIS*_: rank by marginal correlation

^b^ *R*_*SKI*_: rank by prior knowledge integrated

## Discussion and conclusions

In a typical omics study such as gene expression analysis or GWAS, a common scenario is that first a candidate list is generated based on some statistical test procedures (e.g., *t*-test for case-control study), and biomarkers are selected for downstream analysis or validation based on expert domain knowledge. In this study, we developed a variable selection framework, screening with prior knowledge integration (SKI), to integrate two steps into one statistical framework. Inspired by sure independence screening (SIS) method, we break the procedure into two stages: first a geometric average combining the marginal information and external information together is used first to reduce the huge number of parameters to a relative small number; and then a more sophisticated methods such as LASSO are used to refine the model.

The rationale of SKI is to increase the sample size while limiting the noise by selecting a proper *α*. Incorporating external knowledge could lead to more stable results since the prior knowledge is drawn from long-time accumulated studies, and thus rescue the signals overwhelmed by random artifacts in the data at hand. The knowledge relevance is evaluated by carefully selecting *α* to avoid arbitrariness. The similar idea could be found in machine learning techniques such as weighted ensemble predictors [36].

The proposed approach is general and is not limited to any specific type of prior knowledge as long as the variables could be ranked based on some external criteria. In this study, we showed an application example in drug response prediction. Since the only input for our method is a pre-ranked feature list, it could be easily modified to accommodate other applications. Though, the method was developed for knowledge integration, it is suitable for data integration. In our simulation, we showed if the data heterogeneity is strong, the performance of the proposed method is even better than analysis by dataset pooling.

Bergersen et al. has proposed a weighted LASSO (wLASSO) procedure with data integration, which shared a similar idea of our approach [37]. However, there are three major differences between SKI and wLASSO. First, wLASSO incorporates the external information in the penalty terms of LASSO, making it similar to adaptive LASSO. Users have to carefully select the weight terms since it will affect the model fitting directly. Our rank based method is introduced in the screening procedure; it only promotes variables into the model, but will not affect the final model fitting. Second, our approach is more general for knowledge integration. It is difficult to generate a weight function for some abstract biological and medical knowledge, but it is always feasible to give a priority. Finally and the most importantly, one of the purposes to design sure independence screening is to accelerate the data analysis. The computing of complexity is *O(np)* smaller than LASSO’s complexity, which is *O(npmin{p,n})*. SKI enjoys the same advantage as SIS in terms of low computing complexity when dealing with ultra-high dimensional datasets.

SIS has extended to more generalized fields such as generalized linear models, additive models, cox models, and model-free feature selections. In this study, we only discuss the linear and generalized linear model. But, as a screening-based method, SKI is apparently flexible to extend to more generalized fields, too. On the other hand, Li et al. proposed a variant methods, robust rank correlation screening (RRCS) method, which is based on the Kendall τ correlation coefficient between response and predictor variables rather than the Pearson correlation of SIS [38]. They showed the RRCS procedure could be more robust against outliers and influence points in the observations. It is also feasible for us to implement an RRCS-based SKI by replacing the Pearson marginal correlation by Kendall’s marginal correlation, which will be the focus of future work.
